# Recurrence of Acute Page Kidney in a Renal Transplant Allograft

**DOI:** 10.1155/2016/3898307

**Published:** 2016-09-20

**Authors:** Rajan Kapoor, Carlos Zayas, Laura Mulloy, Muralidharan Jagadeesan

**Affiliations:** ^1^Department of Transplant Nephrology, Augusta University Medical Center, Augusta, GA, USA; ^2^Department of Transplant Nephrology, George Washington University Hospital, Washington, DC, USA

## Abstract

Acute Page Kidney (APK) phenomenon is a rare cause of secondary hypertension, mediated by activation of renin-angiotensin-aldosterone system (RAAS). Timely intervention is of great importance to prevent any end organ damage from hypertension. We present a unique case of three episodes of APK in the same renal transplant allograft.

## 1. Case

**Figure 1:**
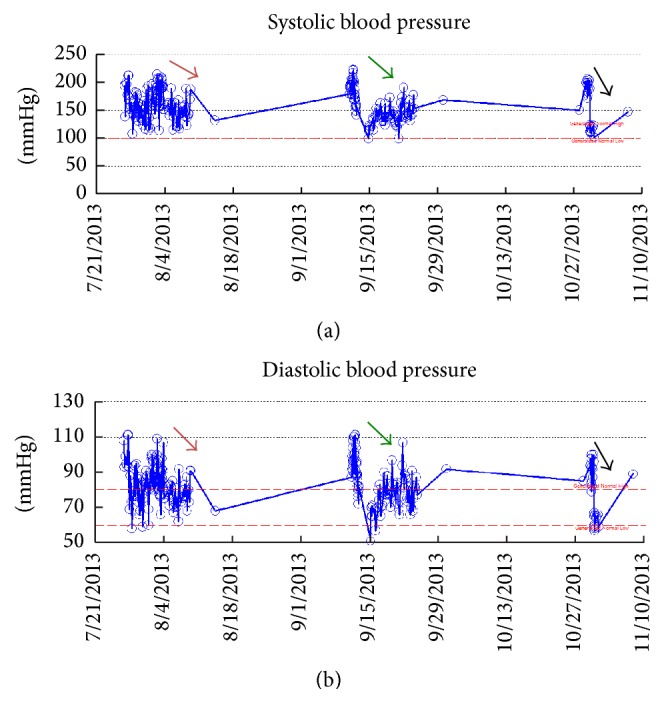
Data points showing systolic blood pressures (a) and diastolic blood pressures (b). Different colored arrows show different hospital admissions. The arrows depict the improvements in blood pressures after treatment of Acute Page Kidney.

A 42-year-old female with hypertension (HTN), ESRD, secondary to IgA nephropathy and a history of deceased donor renal transplant, presented with acute kidney injury (AKI). Her AKI was presumed to be prerenal due to ongoing gastroenteritis. Her S Cr was elevated to 3.3 mg/dL (from baseline of 2–2.2 mg/dL) and admission blood pressure (BP) ranged with 190/100–210/110 mmHg. She was on mycophenolic acid 360 mg QID, sirolimus 1 mg daily, prednisone 5 mg daily, and carvedilol 50 mg BID. Her renal transplant ultrasonography (USG) showed normal resistive indices (RI) with no hydronephrosis. An allograft biopsy was performed to rule out transplant rejection, as her S Cr continued to be elevated at 3.3 mg/dL even after 72 hours of hydration and negative GI workup. Her antihypertensive regime was adjusted with the addition of nifedipine, clonidine, and minoxidil and her blood pressure ranged within 140–160/80–95 during 48 hours prior to procedure. On postprocedure day 1, her blood pressure was again elevated (170–210/90–110 mmHg) and she had a noticeable drop in her hemoglobin (Hgb) from 10 g/dL to 8.5 g/dL. Her CT scans showed large subcapsular hematoma. Renal allograft angiography failed to show any active bleeding, an AV fistula, or any pseudoaneurysm formation. On postbiopsy day 2, her BP remained high and the S Cr peaked at 4.2 mg/dL prompting the drainage of subcapsular hematoma and a Jackson-Pratt (J/P) drain placement for continuous drainage. This resulted in significant improvement in blood pressure (Figure 1) and her IV antihypertensives were tapered off within 24 hours of drainage. Her S Cr improved to 3.1 mg/dL and her J/P drain was removed on day 5. She was soon discharged on PO antihypertensive regime. Her allograft biopsy showed moderate fibrosis with no evidence of antibody or cell mediated rejection or any other acute process.

Unfortunately, six weeks later she was readmitted with severe anemia (Hgb 6.4 g/dL), AKI (S Cr of 8.3 mg/dL), and hypertensive emergency (BP range of 190/90–210/110 mmHg). Her CT scans confirmed recurrence of the subcapsular hematoma. Again, renal allograft angiography failed to show any active bleeding or any AV malformations. This time, the hematoma was surgically drained to ensure the bleeding stopped after drainage. A J/P drain was again placed which was removed after 2 weeks. She required four sessions of hemodialysis treatments for this episode of AKI but her renal function stabilized at discharge with S Cr of 3.1 mg/dL and improved BP control (Figure 1). A repeat USG at discharge showed complete resolution of hematoma. An overt cause for recurrence of hematoma was not elucidated by angiography. She was not on any anticoagulation and her coagulation profile was normal.

She was admitted again seven weeks later, for a large perinephric seroma with uncontrolled HTN (190/90–200/110 mmHg). The collection was drained emergently with an immediate and significant improvement in her BP (range 110–130/60–70) (Figure 1). Her renal function remained at baseline and she had no acute kidney injury. She had no more recurrence of Page Kidney phenomenon and her antihypertensive regime is managed successfully as an outpatient.

## 2. Discussion

The phenomenon of worsening of blood pressure from compression of the renal parenchyma by either a subcapsular collection or extrarenal collection causing renal hypoperfusion/ischemia and triggering of the renin-angiotensin-aldosterone system (RAAS) is known as Page Kidney. Dr. Page first described it in 1939 [1] when he wrapped a canine kidney with cellophane, causing constrictive perinephritis leading to compression of the kidney parenchyma and hypertension. Along with HTN, AKI can also be seen, more so in the setting of a nonfunctioning contralateral native kidney, a solitary kidney, or a patient with renal allograft. In our patient, uncontrolled HTN and AKI were both seen.

APK most commonly is associated with perinephric hematoma. In one case series [2] blunt trauma was reported as the cause in 44% of patients with APK. In the present world, where renal biopsies are done extensively, many more cases of perinephric hematoma causing APK have been described in the postrenal biopsy setting, both after native and transplant kidney biopsy. It holds more importance in the case of transplant kidney as missing the diagnosis and delayed intervention might lead to graft loss. The incidence of APK posttransplant kidney biopsy has been reported around 1% by Chung et al. [3], reported less than 4% by McCune et al. [2], and more recently reported around 36% by Dopson et al. [4]. Apart from perinephric hematoma, authors have suggested APK with seroma or urinoma [2, 5], with postsurgical lymphocele [6], perinephric tumors [7], and postextracorporeal shock wave lithotripsy [8]. It has also been described by spontaneous hematoma in a patient with polyarteritis nodosa [9]. Our case of multiple recurrences of APK episodes in the same allograft is the first reported case of its kind. The first episode of APK was due to subcapsular bleed after the biopsy. The first recurrence was presumed to be due to very slow continuous oozing of blood in the subcapsular space due to platelet dysfunction given patient's recent AKI on CKD stage 4 status [10]. The second recurrence of APK was due to perinephric seroma that formed after open surgical drainage of the hematoma during previous admission. During the first two episodes of APK, renal transplant angiography failed to show any active bleeding source. Our patients' coagulation profile was normal and was not on any form of anticoagulation. There was no clinical suspicion of any form of vasculitis and the biopsy also showed no active vasculitic process. Chung et al. [3] described it in 4 patients (out of 518 transplant kidney biopsies); all 4 cases presented with AKI secondary to subcapsular hematoma in the postbiopsy setting. Acute decompression led to improvement in AKI in 3 patients whereas 4th patient lost the graft (acute antibody mediated rejection). Cromie et al. [11] described Page Kidney following living related renal transplantation that presented with refractory hypertension from perinephric hematoma. Successful evacuation by capsulotomy resulted in improved HTN and normal renal function. Vanwalleghem et al. [6] presented peritransplant lymphocele as a cause of Page Kidney. Yussim et al. [12] described successful treatment of Page Kidney by removal of fibrotic perinephric tissue that developed as a consequence of surgical treatment of peritransplant lymphocele. All such case reports from transplant literature present good outcomes after primary medical or surgical management. Our case is unique in the fact that even after initial appropriate management and stabilization of first episode of Page Kidney the APK phenomenon recurred again causing even worse symptoms of HTN and AKI with hemodialysis requirement temporarily.

The management of APK and treatment need to be individualized to case-by-case basis and should be guided by extent of organ damage and severity of presentation. In cases with HTN emergency, a more aggressive approach including surgical drainage or arterial embolization [13] may be required. In kidney transplant patients, aggressive management is often necessary to ensure viability of the allograft. In our case, during the first episode, even after 72 hours of aggressive medical management, subsequent interventional radiology management was warranted for resistant HTN and worsening renal function. During second episode even after immediate open surgical evacuation patient remained dialysis dependent for 1 week but eventually recovered renal function. Urgent drainage of perinephric seroma at third presentation resulted in immediate improvement in BP.

In conclusion, APK is a rare but very serious complication that can be seen after renal allograft biopsy. New onset of acute pain over the graft, uncontrolled blood pressure, and reduction of Hgb could be the ominous signs. High risk patients such as patients with coagulopathy and uncontrolled HTN should be followed up closely after APK episode as recurrence of hematoma and new perinephric collections including seroma are possible. Our case report is the first case of Recurrent Acute Page Kidney phenomenon in the same allograft and emphasizes the need for close follow-up of such patients even after initial management.

## Abbreviations

AKI: Acute kidney Injury
APK: Acute Page Kidney
RAAS: Renin-angiotensin-aldosterone system
HTN: Hypertension.

## References

[1] Page I. H. (1939). The production of persistent arterial hypertension by cellophane perinephritis. *The Journal of the American Medical Association*.

[2] McCune T. R., Stone W. J., Breyer J. A. (1991). Page kidney: case report and review of the literature. *American Journal of Kidney Diseases*.

[3] Chung J., Caumartin Y., Warren J., Luke P. P. W. (2008). Acute page kidney following renal allograft biopsy: a complication requiring early recognition and treatment. *American Journal of Transplantation*.

[4] Dopson S. J., Jayakumar S., Velez J. C. Q. (2009). Page kidney as a rare cause of hypertension: case report and review of the literature. *American Journal of Kidney Diseases*.

[5] Haydar A., Bakri R. S., Prime M., Goldsmith D. J. A. (2003). Page kidney—a review of the literature. *Journal of Nephrology*.

[6] Vanwalleghem J., Coosemans W., Raat H., Waer M., Vanrenterghem Y. (1997). Peritransplant lymphocele causing arterial hypertension by a Page kidney phenomenon. *Nephrology Dialysis Transplantation*.

[7] Enakpene E., Janga K., Greenberg S. (2012). Angiomyolipoma rupture causing page kidney in a uremic patient. *Clinical Geriatrics*.

[8] Mathew A., Brahmbhatt B., Rajesh R., Kurian G., Unni V. N. (2009). Page kidney. *Indian Journal of Nephrology*.

[9] Pintar T. J., Zimmerman S. (1998). Hyperreninemic hypertension secondary to a subcapsular perinephric hematoma in a patient with polyarteritis nodosa. *American Journal of Kidney Diseases*.

[10] Lutz J., Menke J., Sollinger D., Schinzel H., Thürmel K. (2014). Haemostasis in chronic kidney disease. *Nephrology Dialysis Transplantation*.

[11] Cromie W. J., Jordan M. H., Leapman S. B. (1976). Pseudorejection: the Page kidney phenomenon in renal allografts. *The Journal of Urology*.

[12] Yussim A., Shmuely D., Levy J., Servadio C., Shapira Z. (1988). Page kidney phenomenon in kidney allograft following peritransplant lymphocele. *Urology*.

[13] Lamarche J., Peguero A., Courville C. (2012). Page kidney successfully treated with intrarenal artery embolization. *Federal Practitioner*.

